# Recent Advances in Magnetite Nanoparticle Functionalization for Nanomedicine

**DOI:** 10.3390/nano9121791

**Published:** 2019-12-16

**Authors:** Roxana Cristina Popescu, Ecaterina Andronescu, Bogdan Stefan Vasile

**Affiliations:** 1 National Research Center for Micro and Nanomaterials, Department of Science and Oxide Materials and Nanomaterials, Politehnica University of Bucharest, 060042 Bucharest, Romania; roxpopescu@yahoo.co.uk (R.C.P.); ecaterina.andronescu@upb.ro (E.A.); 2 Department of Life and Environmental Physics, “Horia Hulubei” National Institute for Physics and Nuclear Engineering, 077125 Magurele, Romania

**Keywords:** magnetite nanoparticles, Fe_3_O_4_, functionalization, surface modification, conjugation, nanomedicine, biocompatibility, clinical translation

## Abstract

Functionalization of nanomaterials can enhance and modulate their properties and behaviour, enabling characteristics suitable for medical applications. Magnetite (Fe_3_O_4_) nanoparticles are one of the most popular types of nanomaterials used in this field, and many technologies being already translated in clinical practice. This article makes a summary of the surface modification and functionalization approaches presented lately in the scientific literature for improving or modulating magnetite nanoparticles for their applications in nanomedicine.

## 1. Introduction

As a preponderance of biological processes begin and take place at molecular level, it is understandable why diagnosis and therapeutic solutions have been sought at the nanoscale. The use of nanoparticles in medicine is determined by the processes occurring at the bio-interface. In this context, manipulation of surface properties is highly important as it can determine the fate and functionality of the nano-system and can be achieved through the application of different surface functionalization.

During the last few years, magnetite (Fe_3_O_4_) nanoparticles have been attracting interest, especially in the area of clinical-oriented medical applications, many of which have already been approved by Food and Drug Administration (FDA), such as diagnosis [1,2], hyperthermia cancer treatment [3] or combating iron deficiencies [4]. This was possible due to their properties like biocompatibility [5,6,7,8], biodegradability [9,10,11], magnetic behaviour [12,13] and the possibility of easy functionalization [14,15]. Other possible uses of these nanoparticles might be in fields like catalysis [16,17], environmental remediation [18,19,20], electronics [21,22,23].

The route of synthesis enables controlling not only the chemical composition, but also the size, shape, surface properties and magnetic properties. The chemical methods for synthesis offer the advantage that the resulting nanoparticles can be functionalized at the end of the process, which ensures improved stability compared to non-functionalized materials and conservation of magnetic properties.

One of the most common and easiest chemical methods for magnetite nanoparticles synthesis is the co-precipitation developed by Massart in 1981 [24]. The method resides in the reaction between the ferric and ferrous ions in a basic medium. Different ferric and ferrous salts can be used as precursors (like chlorides, sulfates) and different bases, such as sodium hydroxide [25,26], ammonia [27,28]. The molar ratio of the precursor ions is usually 2:1 (Fe(III): Fe(II)), however, smaller ratios can be employed (such as 1.5:1), as the oxidation of Fe^2+^ can occur [29] and the pH of the precipitation solution should be kept between pH = 9–14 [26,30]. Also, a low concentration of O_2_ is favorable, in order to prevent the oxidation of the nanoparticles and loss of magnetic properties [27]. A non-oxidant medium can be assured by the addition of nitrogen, in gas form or dissolved (such as in ammonia solution). Typically, the synthesis is undertaken in low-heat conditions (about 80 °C [31]), however, room temperature reactions can take place [32]. Moreover, the introduction of surfactants or other organic molecules in the reaction medium (the precipitation base) or in the precursor mixture, can influence the size, shape and surface properties of the resulting nanoparticles [33,34] through the formation of small micelles which limit the space of nucleation and growth available for the nanoparticle. Interactions between the torganic phase and the terminal groups of the nanoparticles might be facilitated and in situ conjugations of the magnetite nanoparticles can take place [35].

The advantages of the co-precipitation method are rapidity, ease, reproducibility and high-yield synthesis, however, the main disadvantage is given by the fact that, in order to obtain a narrow size distribution of the resulting nanoparticles, some reaction parameters must be strictly assured [36]. Table 1 summarized how reaction parameters influence the properties of the resulting nanoparticles in the co-precipitation of the ferric and ferrous ions.

The solvothermal method is the second most popular method for the obtaining of magnetite nanoparticles and is performed in the presence of solvents, using temperatures that are higher than the boiling points of the solvents. The reaction is performed inside an enclosed system, like the autoclave, at high pressures. The composition of solvents influences the shape and size of the nanoparticles [48] however, the size is significantly determined by the temperature and duration of reactions. Different mixtures of agents such as tri ethylene glycol [49], oleylamine and ethylene glycol [50], or benzyl ether [51]. can be added in the solvent mixture in order to act as reducing agents for the precursor(s), leading to the synthesis of highly stable functionalized magnetite nanoparticles.

The hydrothermal method is based on the use of high temperatures and pressures to obtain single Fe_3_O_4_ crystals [52]. Saturation of the precursors is required to initiate crystallization and this is enabled by a temperature difference between the precursors (crystallization area) and an aqueous area in the autoclave.

The microemulsion method uses micelles as nanoreactors for the nucleation and growth of magnetite nanoparticles in a limited space [53]. Thus, one main advantage of this method would be low polydispersity indices of the resulting nanoparticles and controlled morphology of these. Moreover, the nanoparticles are in situ functionalized through encapsulation [54,55].

Lately, a lot on non-conventional methods have been used in order to obtain magnetite nanoparticles. For example, the gas flame synthesis leads to highly dispersed nanoparticles with low polydispersity indices being obtained [56,57]; moreover in situ functionalization can be applied [58].

A rigorous control of the parameters of the synthesis method leads to crystalline nanoparticles with unique mineralogical phase composition being obtained. Magnetite nanoparticles have inverse spinel structure, with a face centred cubic lattice, where the iron ions are placed in the interstitial sites.

Moreover, a controlled synthesis assures and conserves the native properties of magnetite nanoparticles, such as the property of superparamagnetism, with high magnetic susceptibility, which in the absence of magnetic field shows null magnetization [59,60]. Temperature can randomly change the orientation of the magnetic spins, but this effect can also occur after a certain time (Neel relaxation time), due to the magnetic anisotropy of the nanoparticle. Placing Fe_3_O_4_ nanoparticles in an exterior magnetic field causes the orientation of the nanoparticles magnetic moments with the magnetic field, while alternated magnetic fields repeatedly change the orientation of the magnetic moments, with an energy loss, converted to thermal energy. In order to preserve the magnetic property of Fe_3_O_4_ nanoparticles, different functionalization approaches are employed.

The fate of magnetite nanoparticles in the human body is highly dependent on size, surface properties and terminal functional groups. It has been proved that the physical characteristics of the nanoparticles, such as size [61,62,63] and shape [64,65,66,67], influence their relationship with living cells. Additionally, surface properties [68,69], not only dictate the interaction with the biological barriers (membranes, vascular lumens), but can also modulate the way in which the nano-complex is perceived by the cells and tissues. In nanomedicine, this can dictate the effectiveness towards clinical translation. A rigorous control of the physical and chemical properties of magnetite nanoparticles can, most of the time, decide the fate of the nano-system and its ability to fulfil the requirements for which it has been designed and developed [70]. The route of administration can also determine the outcome of the nanoparticles, as they can encounter more or less biological barriers in their way to the targeted area.

Ma et al. [71] made a study on Kumming mice that were daily injected intraperitoneally during 1 week with different concentrations of Fe_3_O_4_ nanoparticles (0, 5, 10, 20, 40 mg/kg), the subjects presenting lesions and the impairment of the hepatic and renal tissues, by means of oxidative mechanisms; the maximum recommended dose was 5 mg/kg. Wang et al. [72] determined the presence of Fe_3_O_4_ nanoparticles in the brain after the intraperitoneal injection. Following intragastric administration of 600 mg/kg magnetite nanoparticles to mice [73], a maximum of concentration was determined in lungs and kidneys after 6 h of administration, in liver, brain, stomach and small intestine after 24 h, in heart and spleen after 3 days, respectively in peripheral blood after 5 days. Intravenous injection (15 mg/kg, 5 times) in C57BL/6 mice determined an accumulation of magnetite nanoparticles in liver, lungs and spleen, which were degraded to non-magnetic iron oxide species [74].

**Figure 1:**
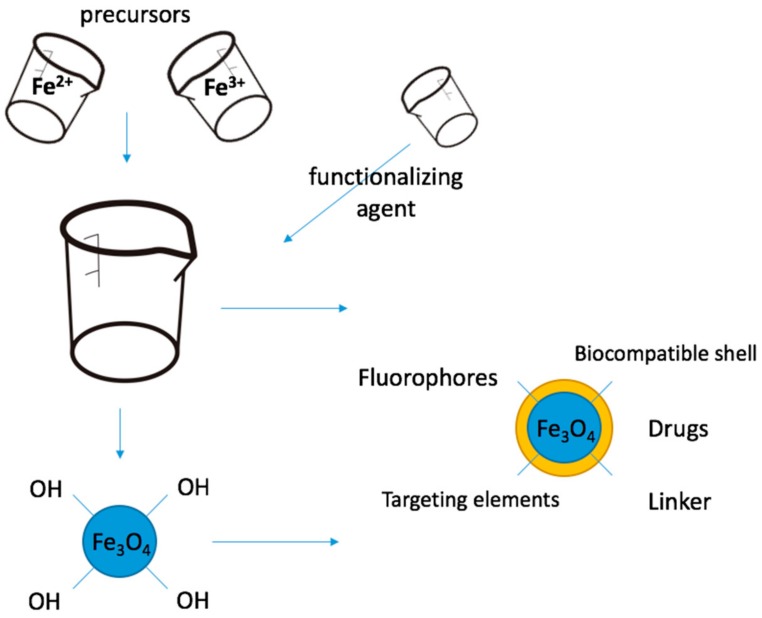
Schematic representation of the two main types of magnetite nanoparticle functionalization processes for medical applications: in situ, respectively, post-synthesis functionalization.

Due to the high surface-to-volume ratio, as a result of the nanometric dimension many hydroxyl terminal groups are available for conjugation with other molecules (Figure 1). It is this property that enables a lot of practicable approaches for surface modification, in order to alter and modulate the physical and chemical behaviour of magnetite nanoparticles. This review article discusses different approaches of functionalization for magnetite nanoparticles applications in medicine.

## 2. Functionalization of Magnetite Nanoparticles

Besides their advantages, magnetite nanoparticles have some major flaws, like rapid agglomeration, chemical reactivity, high surface energy, oxidation, which might alter their biocompatibility, properties and performance. In order to prevent these unwanted events, different surface functionalization is applied.

The functionalization refers to the conjugation of different molecules. In case of nanoparticles, this process determines a modification of the surface chemistry, which leads to changes in the physical, chemical and biological properties.

There are different types of functionalization. Depending on the time when it is done, the functionalization process can be in situ [75,76,77], in case the conjugation takes place simultaneously with the nucleation process of the nanoparticle, during the synthesis or post-synthesis [78,79], when the functionalization reaction(s) is (are) done after the synthesis of the nanoparticles (Figure 1).

By taking into consideration the chemistry of functionalization, either non-covalent or covalent bindings can take place between the surface modifying molecule and the magnetite nanoparticles. The non-covalent conjugation [80,81,82] mainly takes place through interactions that are based on the receptor-ligand affinity principle. Some examples are the electrostatic interactions, entrapment into secondary elements (like polymeric films) or π-π stacking. In this case, mostly ionic bonds appear, following the transfer of one electron from a metallic to a non-metallic atom and the electrostatic interaction between the resulting ions.

In the case of covalent binding [83,84], different chemical reactions can take place during the functionalization process, such as substitution (nucleophile or electrophile), addition (nucleophile or electrophile), elimination, oxidation, reduction, polymerization, or esterification, in presence of different catalysts. In order to conjugate the desired molecule on the surface of the magnetite nanoparticles, intermediary linkers can be used, such as oleic acid [85], aminoproliltriethoxy silane [86], 3-(trimethoxysilyl) propyl methacrylte [87].

Sometimes, the preferred approach is to have a non-specific physical sorption, which would give a less stable conjugation (in case of delivery application or to facilitate the degradation of the nano-system), but chemical sorption can also be employed. In this case, covalent bonds can appear between identical atoms or different atoms which share electrons, each atom participating with one electron. This appears for non-metal elements. These are classified as non-polar covalent bonds (between the same type of atoms), covalent bonds between different atoms, coordinative bonds (when two electrons are shared).

Metallic bonds are chemical bonds that form between metal elements. It is very rare that this interaction takes place between the Fe atoms in the oxide structure of magnetite and other metals, when developing core-shell metallic nanoparticles. For example, Han C.W. et al. [88] has obtained Fe_3_O_4_-Au core-shell nanoparticles by in situ vacuum annealing of dumbbell-like Au-Fe_3_O_4_ nanoparticles obtained by epitaxial growth of magnetite on Au nanoparticles. The process was undertaken using a transmission electron microscope and recorded. During the annealing, the gold nanoparticles transformed into a gold nano-film, which was melting the surface of the magnetite nanoparticle, simultaneously with the reduction of the Fe_3_O_4_ nanoparticle, taking place a strong metal-support bonding between the two components.

Different approaches of magnetite nanoparticles functionalization (Figure 1) will be discussed in the following sections, depending on the type of conjugation agent (inorganic or organic) and related to their biomedical applications.

## 3. Inorganic Functionalization of Magnetite Nanoparticles

### 3.1. Oxides

Among oxides, SiO_2_ (silica) coating is one of the most commonly used approaches for nanoparticle surface modification, especially in the case of iron oxides like magnetite. This is mainly determined by the properties induced by silica coating of Fe_3_O_4_ nanoparticles, such as reducing the aggregation phenomena and thus improving the stability of the resulting functionalized nanoparticles [89], but also enhancing their biocompatibility [65,90].

There are several methods that can be used for SiO_2_ conjugation on magnetite nanoparticles. The most frequently encountered approach is the sol-gel (Stoeber) method, which is based on the hydrolysis of tetraethoxysilane (TEOS) in an alcoholic medium, using ammonia as catalyst [91,92]. The method is popular due to its ease, but also due to the ability to obtain monodisperse-coated nanoparticles, with controlled dimension and shape. By using this approach, the chemical composition and structure, as well as magnetic properties of the Fe_3_O_4_ nanoparticles, are preserved.

Another precise, but more elaborate method for the obtaining of Fe_3_O_4_@SiO_2_ nanoparticles is the microemulsion method, which can be either water-in-oil (W/O) or oil-in-water (O/W). Such methods are usually employed for obtaining of Fe_3_O_4_ nanoparticles and the in situ functionalization [93]. This method can also be microwave assisted [93,94].

Mesoporous silica, such as MCM-41 or SBA-15 have grown in interest due to their biocompatibility [95,96,97] and highly controlled porosity [98,99,100], which enable their use as controlled drug delivery platforms [101,102]. In order to obtain mesoporous silica-coated magnetite nanoparticles, a similar approach as in Fe_3_O_4_@ amorphous SiO_2_ can be employed, but additionally an organic agent is used as template for the pore structure [103,104,105]. Such agents can be cetyltrimethylammonium bromide (CTAB), cetyltrimethylammonium chloride, n-octylamine, tetrapropylammonium bromide (TPABr) [106], triblock polymers like (EO)x-(PO)y-(EO)x (Pluronic L101, P103, P104, P105, F108) [107].

Due to their high porosity, the mesoporous silica-coated magnetite nanoparticles can absorb high quantities of therapeutic agents. Moreover, SiO_2_ is dissolved in acidic environment, such as in the tumor microenvironment, inflammation, bacterial biofilm, or the endo-lysosomal compartments of the cells, making silica-functionalized Fe_3_O_4_ great stimuli-responsive materials for the controlled delivery of therapeutic agents [108,109,110].

Other Si-based molecules have been used as functionalization agents for magnetite nanoparticles, in order to increase their stability or be used as linkers for further surface conjugation. Some examples are (3-aminopropyl)triethoxysilane (APTES) [111,112,113], 3-Aminopropyltrimethoxysilane (APTS) [114], (3-Mercaptopropyl)trimethoxysilane (MPTS) [115], triethoxy vinyl silane (VTES) [116], aminosilane [117,118]. Table 2 summarizes some recent examples of Fe3O4@SiO2 nano-systems and their applications in biomedicine.

Numerous metal oxides have been used as functionalizing agents to modify the surface of magnetite nanoparticles, in order to obtain composites with improved functions. ZnO-conjugated Fe_3_O_4_ nanoparticles have been developed in order to implement photocatalytic properties to the developed nano-systems. This phenomenon appears due to high oxygen vacancies on the surface of the nanoparticles and due to the fact that the electron-hole pairs induced by photon-triggering are inhibited by Fe^3+^ ions [125]. Similar photocatalytic effects are given by Fe_3_O_4_@TiO_2_ nanoparticles [102]. Shi L et al. [31] obtained Fe_3_O_4_@TiO_2_ core-shell nanoparticles using post-synthesis functionalizing based on a hydrothermal approach. Similarly, Zhang L et al. [126] and Choi K-H et al. [127] used the solvothermal synthesis for microsphere preparation.

### 3.2. Metals

The surface conjugation of Fe_3_O_4_ with different metals has been employed in order to improve the biocompatibility of magnetite nanoparticles and to induce an inert character to the final nano-structure. The metal coating of Fe_3_O_4_ nanoparticle surface can be done either directly or through an intermediate functionalizing layer.

Core-shell magnetite@gold nanoparticles are interesting for their multifunctionality. The direct route to obtain this type of nano-composites is by directly reducing Au^3+^ ions on the surface of the Fe_3_O_4_ nanoparticles, using reducing agents such as sodium citrate [60,128], sodium borohydride [129], and hydroxylamine hydrochloride [130]. Through this method mostly result dumbbell-like, core-satellite, or sometimes star-shaped structures, but core-shell nanoparticles can only result after multiple repetitions of the coating procedure. The main disadvantage of this method is the low yield synthesis, as many gold nanoparticles result [131]. Moreover, the reduction of Au^3+^ into Au^0^ takes place at the boiling point of the watery solution (80–90 °C), which might lead to an oxidation of Fe_3_O_4_ and loss of magnetic properties.

Also, a more efficient direct method of conjugation might be in situ functionalization, through the organic synthesis approach [132]. Usually, these routes employ different agents to reduce Fe(acac)_3_ [132] or FeO(OH) [133] in presence of HAuCl_4_ which is simultaneously reduced, forming core-shell structures. Other organic molecules, such as oleic acid [134] are used to act as reducing and stabilization agents at the same time.

The use of an intermediary layer between the previously-synthesized magnetite nanoparticles and the gold layer acts as a “glue” between the two components. In situ or post-synthesis functionalization of iron oxide nanoparticles is undertaken, in order to obtain a functional layer that can either attract the Au^3+^ ions, which are afterwards reduced to Au^0^ using a third substance [135,136], or the conjugated molecules act as a reducing agent themselves [134].

**Figure 2:**
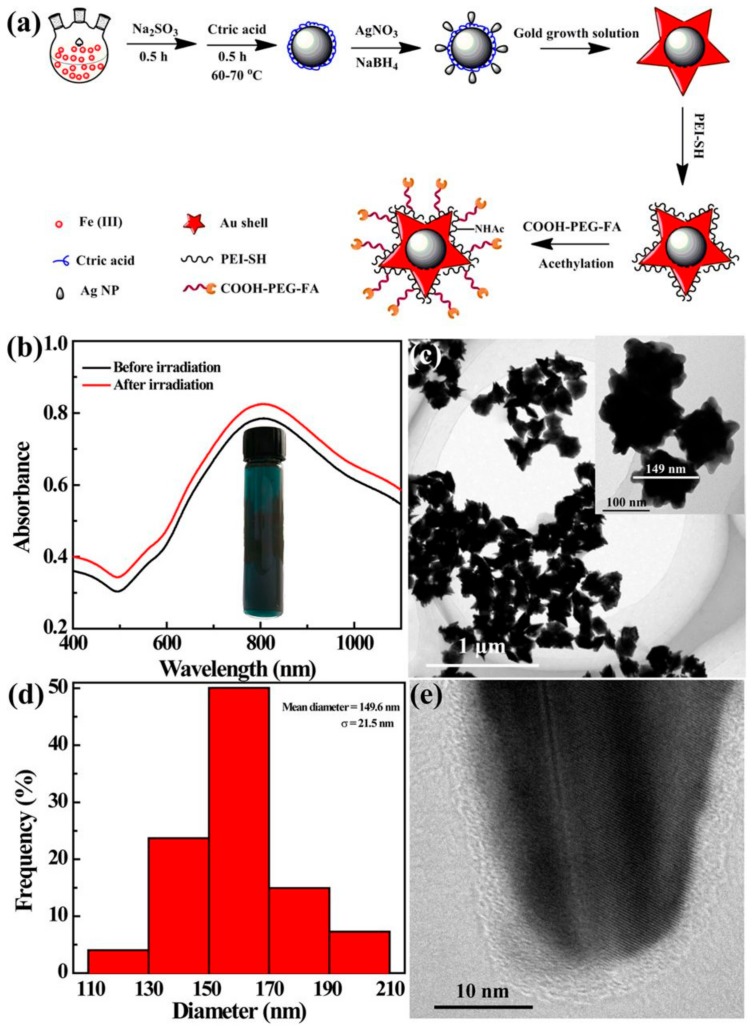
Star-shaped gold-conjugated Fe_3_O_4_ nanoparticles; functionalization with organic molecules (polyethyleneimine, PEI): (**a**) schematic representation of the synthesis and conjugation processes; (**b**) ultraviolet–visible (UV–VIS) spectra for (non-) irradiated the nano-constructs; (**c**) transmission electron micrograph (TEM) of the resulted nanoconstructs; (**d**) histogram distribution of size; (**e**) high-resolution TEM (HR-TEM) of the resulted nanoconstructs; reprinted from [137].

Fe_3_O_4_-Au conjugated nanoparticles have applications in medical imaging. Due to the presence and properties of both magnetite and gold phases, such nanoparticles can be used as a contrast substance in both magnetic resonance imaging (MRI), computer tomography (CT) and photoacoustic imaging (PA). For attempt, Hu Y et al. [137] developed Fe_3_O_4_@Au nano-systems starting from Fe_3_O_4_@Ag@citric acid as seeds for Au^3+^. The resulting star-shaped nanoparticles were functionalized with polyethyleneimine (PEI) to improve stability and folic acid to induce the targeting ability (Figure 2). Ge Y et al. [138] used antibody (McAb) cetuximab (C225) conjugation of Fe_3_O_4_@Au to induce targeting ability for glioblastoma. The functionality of Fe_3_O_4_@Au nano-composites for targeted tumor imaging has been proved in vivo [137,138,139].

Other possible application of magnetite-gold nano-conjugates refers to their use in cancer radiotherapy, following their activation with different types of radiation: ionizing radiation (IR) [140,141], near-infrared (NIR) radiation [134,142] and radiofrequency (RF) [143] radiation. Radiotherapy mediated by nanoparticles has been considered as an approach that overcomes the resistance of tumor cells to radiotherapy and/or chemotherapy [144,145,146,147].

Generally, the use of metal elements to radiosensitize tumor cells is based on increasing the photoelectrical absorption, after their accumulation inside the malignant tissue. The high atomic number elements absorb most of the radiation compared to the surrounding healthy tissues and, due to the photoelectric and Compton effects, lower energy photons, Auger secondary electrons and low-energy secondary electrons are released [148,149,150,151]. Also, an enhanced production of reactive oxygen species occurs, due to the formation of secondary electrons and photons, but also due to the high surface reactivity of the nanoparticles [152,153]. This affects directly the DNA of the tumor cells. Moreover, nanoparticles can directly interact with the DNA, forming bonds or intercalating intro the DNA chain [154,155]. The biological outcome is oxidative stress, cell-cycle disruption and DNA repair inhibition [148] in the tumor cells.

The radiofrequency ablation (RFA) as a new method for cancer treatment has recently attracted more interest due to the fact that it does not harm normal tissues, when using frequencies from 10 kHz–900 MHz; the radiation has high penetration capability and non-ionizing effects on the tissues. The mechanism of toxicity upon cancer cells is produced by the induced thermal disruption determined due to the friction appearing in the ionic collisions of the biomolecules, when aligning in the alternating current flow [156]. RF-responsive nanomaterials have been proposed as probes for the treatment procedure, because of their ability to produce heat due to the resistance heating (in conductive materials, such as gold [153]), respectively magnetic heating (in magnetic materials, such as magnetite [157]). Gold-conjugated magnetite nanoparticles are excellent candidates for RFA treatment of cancer [142,158].

Another possible application of gold-conjugated magnetite nanoparticles is biosensing, due to the surface plasmon resonance property of gold [159,160,161,162]. Moreover, further functionalization of Fe_3_O_4_@gold with different antibodies gives the ability of specific targeting of cells, which together with the magnetic properties of the nano-systems enable their applicability in cell sorting or cell separation [163,164].

Platinum-conjugated magnetite nanoparticles also have possible applications in radiotherapy enhancement. Also an inert noble metal, Pt has an atomic number higher than Au, being able to induce higher radiosensitizing effects [165,166]. Ma M. et al. [167] used a “glue” layer, DMSA (meso-2, 3-dimercaptosuccinic acid), for Pt ions that were reduced using NaBH_4_ on the surface of previously-synthesized magnetite nanoparticles, in order to obtain dumbbell-like structures. A similar approach was employed by Wu D et al. [168] who used MnO_2_ as intermediary layer for Pt ions absorption followed by reduction on the surface of the Fe_3_O_4_@MnO_2_ nano-conjugate.

Silver coated magnetite nanoparticles can be obtained using the same approaches as gold-magnetite conjugates. Their applications in the medical field vary from catalysis [169], contrast substance in medical imaging [170,171], radiation therapy [172], the most frequent application being given by their anti-microbial properties [173]. Chang M et al. [174] obtained Fe_3_O_4_@Ag nanoparticles using in situ functionalization and proved their effect against *E. coli* strains. Brollo M. E et al. [175] synthesized brick-like nano-composites using a thermal decomposition method and in situ conjugation.

## 4. Carbon-Based Functionalization of Magnetite Nanoparticles

The carbon-based functionalization of magnetite nanoparticles is treated separately from the (in)organic sections, as both inorganic (such as SiC [176]), as well as organic (graphene, carbon nanotubes) and Fe_3_O_4_@C composites are approached.

The majority of Fe_3_O_4_@C composites applications are in electronics (used as supercapacitors [177], anode materials in lithium-ion batteries [178], absorbents [177]). These materials can be obtained by in situ or post-synthesis functionalization, using the hydrothermal approach [179,180,181].

For applications in the biomedical field, the conjugation of magnetite nanoparticles and carbon-based nanostructures, such as graphene, carbon nanotubes or fullerenes are more often encountered. Amide bonding is a very frequent approach in conjugation of Fe_3_O_4_ and carbon-based nanoparticles [158], alongside with click chemistry. These types of reactions are modular reactions like cycloadditions, nucleophilic ring-openings, carbon multiple bond additions and non-aldol carbonyl reactions [182]. The most common type in functionalizing carbon-based nanomaterials is Cu(I)-catalysed azide-alkyne 3+2 cycloaddition (CuAAC) [183]. Table 3 presents recent exampled of Fe_3_O_4_-carbon nanoparticles conjugates.

## 5. Organic Functionalization of Magnetite Nanoparticles

The functionalization of magnetite nanoparticles with organic compounds is mostly done in order to improve their stability [192] and biocompatibility [193]. Another reason would be to improve their interaction with biological barriers (cellular membranes, vascular endothelium, blood-brain barrier) and facilitate the nanoparticles’ passage through these [194,195].

Furthermore, magnetite nanoparticles have a hydrophobic character which favours the adsorption of serum proteins, causing not only blood clogging, but also leading to the opsonisation phenomenon. Through this, the nanoparticles are immediately collected by the cells of the mononuclear phagocyte system and eliminated from systemic circulation. In order to improve the pharmacological kinetics of the magnetite nanoparticles, functionalization with hydrophilic polymers, such as polyethylene glycol (PEG) [196] is applied.

In case of controlled delivery of therapeutic substances, organic materials and especially polymers are the best stimuli-responsive materials (responsive to changes in temperature, pH, light). Fe_3_O_4_ nanoparticles functionalized with biocompatible responsive polymers are ideal for such applications, as the magnetite core enables magnetic targeting properties of the system, while the soft shell encapsulates large quantities of drug molecules.

Also, polymers enable many available functional groups for the conjugation of other molecules. Thus, specific molecules can be conjugated for targeting certain type of cells or area of the body (like folic acid [197,198], L-3,4- dihydroxyphenylalanine (L-DOPA) [199], riboflavin [200], arginine-glycine-aspartate (RGD) [201] for cancer targeting) and/or light-responsive molecules for detection and imaging (such as fluorescein isotiocianate-FITC [202]). Moreover, Fe_3_O_4_ can be used as contrast substance in MRI because of its ability to alter the spin-spin relaxation time T2 of the surrounding water protons [203]. Given all these properties, functionalized magnetite nanoparticles can be used as multifunctional platforms for cancer detection and therapy.

Organic materials for magnetite nanoparticles functionalization will be discussed in separate sections as follows: small molecules and surfactants, lipids, polymers, phytochemicals, respectively drug molecules.

### 5.1. Small Molecules and Surfactants

Functionalization of magnetite nanoparticles with amphiphilic molecules (surfactants) has been proved as a good solution to improve the stability of the suspensions [204,205]. However, surfactants can rather have a toxic behaviour and are not recommended for biological applications [206,207,208].

Instead, functionalization with small molecules was proposed. Oleic acid is the most common small lipophilic molecules used for the functionalization of magnetite nanoparticles. Fe_3_O_4_@oleic acid has good stability [209], biocompatibility [210] and can be used for further functionalization: oleic acid can act either as a “glue” layer to conjugate other compounds [211] or as a starting point in ligand exchange approach [212,213]. 

Functionalization of magnetite nanoparticles with small molecules or surfactants is mostly done in situ using solvothermal [51,214,215] or microemulsion [53,216] approaches, however, post-synthesis conjugation can also be done [217,218]. 

**Figure 4:**
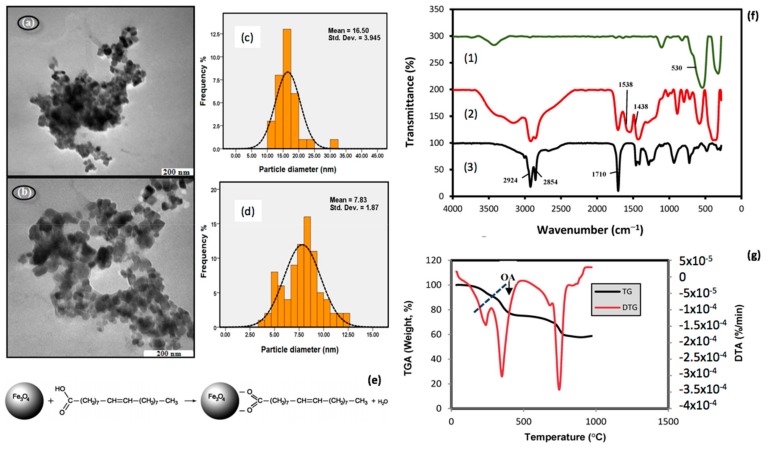
Surface conjugation of magnetite nanoparticles with oleic acid: transmission electron microscopy (TEM) image for (**a**) bare Fe_3_O_4_, respectively (**b**) oleic acid conjugated Fe_3_O_4_; particle diameter distribution for (**c**) bare Fe_3_O_4_, respectively (**d**) oleic acid conjugated Fe_3_O_4_; (**e**) schematic representation of the capping principle; (**f**) Fourier transform infrared (FTIR) spectra of Fe_3_O_4_ (**1**) Fe_3_O_4_/oleic acid (**2**), respectively oleic acid (**3**); (**g**) thermogravimetric analysis (TGA) and differential thermogravimetric analysis (DTA) curves for oleic acid conjugated Fe_3_O_4_; adapted from [219].

Figure 4 [219] illustrates an approach for oleic acid capping of magnetite nanoparticles and the morphological and hydrodynamic properties of the resulting functionalized nanoparticles, in comparison with bare Fe_3_O_4_.

### 5.2. Lipids

Lipids are the main component of cellular membranes, thus conjugation with magnetite nanoparticles would be ideal for biomedical applications. Lipid-coated nanoparticles favour the interaction with and passage through biological membranes [220,221], enhancing the biocompatibility of Fe_3_O_4_ nanoparticles [197,222] and preventing the opsonisation phenomenon [223]. The obtaining of lipid-conjugated magnetite nanoparticles is most of the time done through encapsulation [224,225].

### 5.3. Polymers

The functionalization of magnetite nanoparticles with polymers can be undertaken using both in situ and post-synthesis functionalizing. It is very common in case of co-precipitation method for Fe_3_O_4_ synthesis to introduce polymer molecules in the precipitation solution, in order to determine the simultaneous functionalization, nucleation and growth of the nanoparticles [226,227]. In this case, mostly non-covalent bonds (electrostatic forces) appear between the polymers and magnetite nanoparticles.

The latter method starts from previously synthesized magnetite nanoparticles that can be conjugated with different polymers through the available hydroxyl groups on their surface. These are mostly condensation reactions. One approach is through the ester bond formation. Also, intermediate linkers can be used, such as APTES, which enable amine terminal groups on the surface of the magnetite nanoparticles. These can be then coupled with different polymers through an amide bond formation.

The main reason for polymer surface functionalization of magnetite nanoparticles is the increase of stability, as the polymeric molecules act as splicing agents between the magnetic nanoparticles, preventing their aggregation. The longer the polymeric chain, the higher the stability of the nanoparticles. However, this can produce an inverse effect, as a reduced magnetic response can occur when stimulating the functionalized nanoparticles with an exterior magnetic field.

Polyethylene glycol (PEG) is the most widely used polymer for magnetite nanoparticles functionalization. PEG with different molecular weights are employed, in order to modulate the hydrodynamic properties of the resulting nano-composites and to improve their stability [228,229]. Other frequently used polymers for Fe_3_O_4_ nanoparticles functionalization are polyethyleneimine (PEI) [230,231], glucose [232,233,234], dextran [235,236], and chitosan [237,238,239]. Table 4 summarizes some examples of polymer-functionalized magnetite nanoparticles and their applications.

Maier-Hauff K. group has studied the effects of soft polymer coated Fe_3_O_4_ nanoparticle-mediated hyperthermia combined with external beam radiotherapy on glioblastoma multiforme patients [250,251,252]. Nowadays, this treatment plan has been clinically approved and used by MagForce [3].

Hyperthermia is a therapeutic procedure for cancer which rises the temperature of the tissue to about 41-45^o^C for a certain period of time [253]. Tumor cells are sensitive to these temperatures, while normal healthy cells endure temperatures up to 46–47 °C. Nanoparticle-mediated magnetic hyperthermia uses the magnetic property of Fe_3_O_4_ nanoparticles to produce thermal energy [254]. The nanoparticles are exposed to external alternated magnetic fields which cause successive (de) magnetization, the supplementary energy to reach the relaxation state being converted to thermal energy [255].

### 5.4. Phytochemicals

Phytochemicals are chemical products derived from plants, which might have beneficial effects on human health. Conjugation of magnetite nanoparticles with different phytochemicals was done in order to improve their biocompatibility [256,257] and induce certain therapeutic properties (antibacterial [32,258,259,260], anticancer [11,261]). Mostly, these plant-originated chemicals are used as reducing agents for the iron precursors [262,263] during the synthesis of the nanoparticles. This process enables an in situ functionalization of the resulting materials with molecules in the plant extracts, which are mostly rich in hydroxyl groups. However, post-synthesis functionalization can also be employed [256].

In traditional medicine, phytochemicals have been used extensively due to their potential therapeutic activity, continuing to be the basis of alternative therapeutic approaches even today, in cancer therapy [264,265], anti-microbial applications [258,266], anti-inflammatory approaches [267,268], anti-viral and immune system enhancement [269]. Moreover, folic acid has been used extensively as targeting agent for tumour cells [270,271], as these cells exhibit a higher density of folic acid receptors on the membrane, compared to healthy cells.

**Figure 6:**
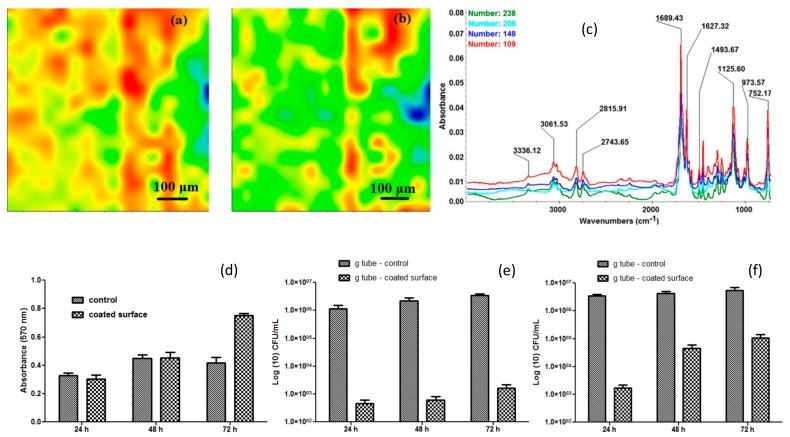
Matrix-assisted pulsed laser evaporation (MAPLE)-deposited Fe_3_O_4_@*Cinnamomum verum* at fluence F = 400 mJ/cm^2^: Infrared microscopy-distribution of intensity of (**a**) 2815 cm^−1^, (**b**) 1689 cm^−1^, (**c**) IR spectra; (**d**) biocompatibility evaluation for endothelial cells; antibacterial evaluation—*S. aureus* biofilm formation (**e**), respectively, *E. coli* biofilm formation (**f**) [32].

In the case of anti-bacterial applications, one important branch refers to combating the medical devices associated infections and biofilm formation, one approach for preventing antibiotic resistant bacteria contamination being the use of alternative medicine. Figure 6 illustrates the compositional structure and biological characterisation of matrix-assisted pulsed laser evaporation (MAPLE) deposited Fe_3_O_4_@*Cinnamomum verum* thin films. These have been developed in the idea of implant surface modification with anti-bacterial potential. Such substrates are biocompatible for eukaryote cells (in the surrounding tissues) and exhibit a toxic effect against prokaryote (bacterial) cells.

### 5.5. Drug Molecules

Magnetite-based nano-systems have been broadly used as drug-delivery systems [272,273,274,275]. A direct conjugation of the drug with the functional groups of magnetite is mostly undertaken in order to assure a targeted transport of the therapeutic molecules at the site of action through magnetic directing. Weak bonding (such as non-covalent interactions) between the two components is preferred, in order to allow facile delivery of the drug. Strong interactions may affect the chemical structure of the drug molecule and determine therapeutic properties loss.

## 6. Conclusions

In the context of the advancement of magnetite nanoparticles implications in nanomedicine, a high control of their hydrodynamic and biocompatibility properties should be guaranteed, besides the fulfilment of their main biomedical function. This can be assured through the conjugation of secondary components. This review summarizes the latest advances in various approaches for Fe_3_O_4_ nanoparticles functionalization for nanomedicine applications:

- Multifunctionality of Fe_3_O_4_ nanoparticles is given by its properties (magnetism, biocompatibility);
- They have many applications in the medical field, among which a few have been approved by the FDA for clinical use (MRI contrast substance, magnetic hyperthermia, iron deficiency supplement);
- The route of synthesis also determines the surface functionality among other properties;
- Surface functionalization determines an alteration of the surface chemistry, leading to changes in the physical, chemical and biological properties;
- Classification of functionalization processes. Depending on: time of functionalization (in situ, respectively post synthesis), chemistry of functionalization (non-covalent and covalent), chemistry of the functionalizing agent (inorganic and organic);
- Non-specific physical sorption is preferred in applications such as drug delivery systems;
- Among the oxides, SiO_2_ coating of magnetite nanoparticles is the most common because it enhances the biocompatibility and stability of the nanoparticles; some common approaches to obtain this conjugation are the sol-gel method, respectively, microemulsion;
- The mesoporous silica coating is biocompatible and offers high controlled porosity; is good for drug delivery applications;
- Metal oxide (ZnO, TiO_2_) functionalization has photocatalytic applications;
- Surface functionalization of magnetite nanoparticles with metals induces an inert character; the most popular approach in this category is the conjugation of Fe_3_O_4_ with gold because of its biocompatibility and multifunctionality; approaches to obtain this type of nanoparticles are: reduction of gold ions on the surface of magnetite nanoparticles, respectively, the organic synthesis approach; the final applications are numerous: medical imaging (MRI, CT, PA), radiosensitiation, radiofrequency ablation, biosensing, cell sorting;
- Carbon-Fe_3_O_4_ nano-composites mostly have applications in electronics, but also in biosensing and drug delivery systems; in order to obtain these materials, the direct precipitation of magnetite nanoparticles on the surface of the carbon nanomaterial can be applied or a hydrothermal approach for in situ functionalization;
- The conjugation of magnetite nanoparticles with organic molecules has the advantage of improving the stability, biocompatibility and interaction with biological membranes of the Fe_3_O_4_; mostly has applications in the development of drug delivery systems;
- Surfactants have been used to improve the stability of the magnetite nano-constructs, but can have toxic effects;
- Lipid-encapsulated nanoparticles enhance the biocompatibility of the magnetite nanoparticles and improve their interaction with biological membranes, while preventing opsonisation;
- The functionalization of Fe_3_O_4_ with polymers is the type of surface modification most encountered for these nanoparticles and can be undertaken both in situ (through electrostatic interactions) or post-synthesis (through condensation); it increases the stability and biocompatibility of magnetite nanoparticles, leading to applications in medical imaging, hyperthermia treatment of cancer, drug delivery systems, tissue engineering;
- A polymer-coated Fe_3_O_4_ nanoparticle (MagForce) has been approved by the FDA for use in hyperthermia treatment of cancer;
- Drug-delivery systems based on magnetite nanoparticles can be developed for commercial medicines or phytochemicals; the therapeutic molecule can be directly conjugated on the Fe_3_O_4_ surface or can be attached through an intermediate layer;
- Phytochemicals-Fe_3_O_4_ are popular alternative medicines with antimicrobial, antitumor, anti-inflammatory or antiviral applications; conjugation with magnetite nanoparticles can be undertaken through both weak and strong interactions;
- Conventional drugs are mostly attached through strong interactions from the magnetite nanoparticles.

## Figures and Tables

**Figure 3:**
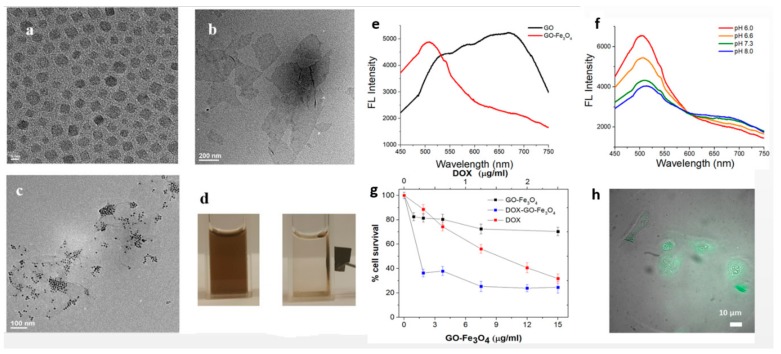
Fe_3_O_4_@(3-aminopropyl)triethoxysilane (APTES)-graphene oxide nano-system for drug delivery and diagnosis in cancer: (**a**) TEM of Fe_3_O_4_ nanoparticles; (**b**) TEM of graphene oxide; (**c**) TEM of Fe_3_O_4_-graphene oxide conjugates; (**d**) magnetic manipulation of Fe_3_O_4_-graphene oxide conjugates in aqueous solution; (**e**) fluorescence specra of graphene oxide and Fe_3_O_4_-graphene oxide conjugates; (**f**) fluorescence specra of Fe_3_O_4_-graphene oxide conjugates at different pH; (**g**) HeLa cell survival (%) after incubation with equivalent concentrations of Fe_3_O_4_-graphene oxide conjugates, Fe_3_O_4_-graphene oxide conjugates loaded with doxorubicin, respectively doxorubicin; (**h**) fluorescence image of internalized Fe_3_O_4_-graphene oxide conjugates in HeLa cells; adapted from [185].

**Figure 5:**
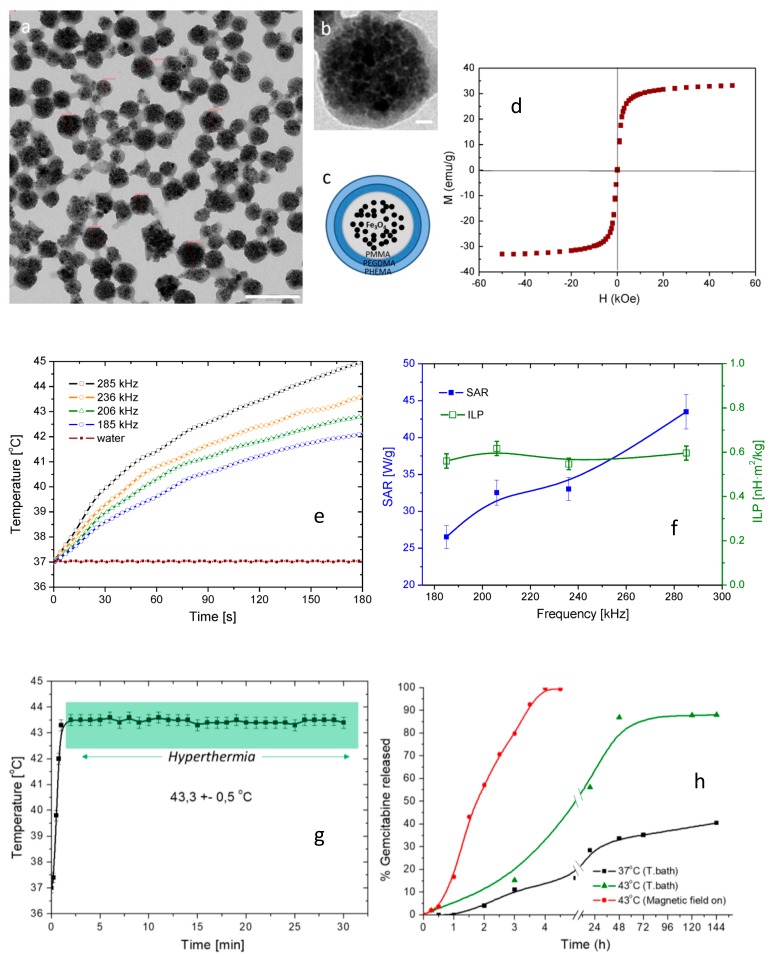
MagP-OH particles: (**a**) TEM image, scale 200 nm, (**b**) TEM detail, scale 20 nm, (**c**) schematic representation of MagP, (**d**) magnetisation curve of MagP, (**e**) time evolution of temperature for various frequencies, (**f**) Specific Absorption Rate (SAR) and Intrinsic Loss Power (ILP) for Ha = 16.2 kA/m, (**g**) hyperthermia measurement, (**h**) drug release measurement; adapted from [242].

**Table 1:** Influence of reaction parameters on the properties of magnetite nanoparticles resulting from the co-precipitation method.

No.	Reaction Parameter	Property	Measure	Reference
1	Fe^3+^/Fe^2+^ ratio	Iron oxide phase	Directly proportional	[37]
		Magnetism	Inversely proportional	[38,39]
		Dimension	Directly proportional	[39,40]
2	pH value	Iron oxide phase	Inversely proportional	[41]
		Magnetism	Inversely proportional	[38,42]
		Dimension	Insignificant	[42]
3	Type of base	Iron oxide phase	Depending on the type of base	[26]
		Magnetism	Depending on the type of base	[26]
		Dimension	Depending on the type of base	[26]
4	Temperature	Iron oxide phase	Directly proportional	[43]
		Magnetism	Inversely proportional	[44]
		Dimension	Inversely proportional	[40,45]
5	Concentration of precursors	Dimension	Directly proportional	[40]
6	pH of the precursor solution	Iron oxide phase		[40]
		Magnetism		[40]
		Dimension	Directly proportional	[40]
7	Addition of surfactants	Dimension	Directly proportional	[38,46,47]
		Surface charge	Dependent on the surfactant	[47]
		Composition	Dependent on the surfactant	[47]
		Shape	Dependent on the surfactant	[33]
		Magnetisation	Dependent of the surfactant	[47]

**Table 2:** Recent approaches in Fe_3_O4-SiO_2_ based nanostructures conjugates.

No.	System Description	Application	Type of Conjugation	Evaluation	Reference
1	Fe_3_O_4_@SiO_2_	Magnetic resonance imaging contrast substance as in vivo stem cell tracker	Negatively charged Fe_3_O_4_@citrate act as seeds for Si precursor; encapsulation using sol gel method;	Determination of distribution and chemical changes dynamics of Fe_3_O_4_@SiO_2_; high chemical stability; distribution in cytoplasm;	[119]
2	Fe_3_O_4_@SiO_2_/anti-rHBsAg (Hepatitis B surface antigen)	Purification of recombinant Hepatitis B for vaccine production;	In situ functionalization; encapsulation using sol gel method;	In vitro isolation of rHBsAg antigen from Pichia pastoris yeast	[120]
3	Fe_3_O_4_@SiO_2_	Plasmid DNA purification	SiCl_4_ cross-linker between Fe_3_O_4_@NH_3_ and (3-aminopropyl)triethoxysilane (APTES); encapsulation using sol gel method;	Efficient in vitro plasmid DNA purification from *E. Coli* DH5a cells	[121]
4	Fe_3_O_4_@boronic acid/mesoporous (m) SiO_2_	Magnetic and pH triggered drug release;	−	Biocompatibility and high uptake in MC3T3-E1 cells; Controlled drug release and good magnetic properties;	[122]
5	Fe_3_O_4_@mSiO_2_/catalase (CAT)	Enzyme protection in catalysis;	Encapsulation in SiO_2_ using TMOS (tetramethoxysilane) functionalization with APTES for CAT conjugation and growth of mSiO_2_ using CTAB as template and TMOS;	Good stability and catalytic activity	[123]
6	Fe_3_O_4_@oleic acid@mSiO_2_/5-Fluorouracil	Drug delivery for cancer therapy;	In situ Fe_3_O_4_@oleic acid were functionalized with CTAB through weak interaction (Van der Waals); hydrolisation of tetraethoxysilane (TEOS) on Fe_3_O_4_/CTAB; encapsulation in mSiO_2_ using the inversed microemulsion method;	In vitro biocompatibility for MCF-7 cells; efficient drug loading;	[124]

**Table 3:** Recent approaches in Fe_3_O_4_-carbon-based nanostructures conjugates.

No.	System Description	Application	Type of Conjugation	Evaluation	Reference
1.	Fe_3_O_4_ @APS–graphene/5-Fluorouracil	Drug-delivery systems for cancer treatment;	Amide bonding using 1-ethyl-3-(3-dimethylaminopropyl) carbodiimide	In vitro drug release at acidic pH; efficient in vitro internalizing in hepatocarcinoma HepG2 cells; biocompatibility of the carrier nanoparticles;	[184]
2.	Fe_3_O_4_@ APTES/graphene oxide (GO)/doxorubicin	Drug-delivery systems and imaging diagnosis in cancer management;	Amide bonding using N-(3-Dimethylaminopropyl)-N′-ethylcarbodiimide hydrochloride (EDC)	In vitro low cytotoxicity compared to GO; superparamegnetic properties and 10.7 r2/r1 relaxivity; fluorescence in VIS; high doxorubicin loading and 2.5 fold higher efficiency; (Figure 3)	[185]
3.	Fe_3_O_4_@azide-sodium ascorbate-GO@ alkyne	Efficient absorbent and removal of dyes;	Click chemistry approach between the azide functional groups on the Fe_3_O_4_, sodium L-ascorbate and alkyne functional groups on GO;	Superparamagnetic properties; efficient absorbent and removal of dyes;	[186]
4.	Fe_3_O_4_@GO	Magnetic fluids;	Absorption;	Improvement of friction and wear performances with magnetic field;	[187]
5.	Polyvinyl alcohol (PVA)/ Fe_3_O_4_@ carbon nanotubes (CNTs)	Absorbent and dye removal; Anti-bacterial effects;	−	Optimal dye removal and anti-bacterial properties;	[188]
6.	Fe_3_O_4_/multi walled CNTs/laser scribed graphene/chitosan/glassy carbon electrode	Detection of heavy metals	−	Electrode for the determination of Cd^2+^ and Pb^2+^ using square wave anodic stripping voltammetry; wide linear range; ultralow detection limit; excellent repeatability, reproducibility, stability;	[189]
7.	Single-walled CNTs-PEG-Fe_3_O_4_@ carbon quantum dots (CQD)/doxorubicin/sgc8c aptamer	Targeted photodynamic and photothermal ablation of tumor cells; controlled drug delivery; targeted imaging using fluorescence and magnetic resonance imaging (MRI)	Through polyethylene glycol (PEG) linker using amide bonding;	Near infrared triggered production of reactive oxygen species and heat; good imaging properties; good biocompatibility of the carrier and cellular internalization; high drug loading ability; selective accumulation at tumor site in human adenocarcinoma (HeLa) tumor-bearing mice intravenously injected with the system;	[190]
8.	GO-Chitosan/Fe_3_O_4_/glucose oxidase	Glucose biosensor and magnetic resonance imaging;	−	Good glucose biosensing ability;	[191]

**Table 4:** Recent approaches in Fe_3_O_4_-polymer-based nanostructures conjugates.

No.	System Description	Application	Type of Conjugation	Evaluation	Reference
1.	Fe_3_O_4_@ poly(polyethylene glycol methacrylate-co-acrylic acid) (P(PEGMA-AA))	Hyperthermia and MRI contrast substance;	Electrostatic interactions between the acrylic acid and positively-charged Fe3O4;	Improved stability and salt tolerance; excellent blood compatibility; formation of blood protein corona; resistance to cell internalization; improvement of contrast in MRI;	[240,241]
2.	Fe_3_O_4_/methyl methacrylate/ethylene glycol dimethacrylate/hydroxyl ethyl methacrylate/gemcitabine	Hyperthermia and drug delivery for cancer therapy	−	Good incorporation of drug; temperature triggered release; (Figure 5)	[242]
3.	Fe_3_O_4_@PEG/Doxorubicin	Drug delivery and hyperthermia in cancer treatment;	In situ conjugation	pH responsive release of drug; no cytotoxicity of Fe_3_O_4_@PEG for human fibroblasts; Fe_3_O_4_@PEG/Doxorubicin showed good internalization and cytotoxicity for mouse skin fibrosarcoma; good magnetic properties;	[243]
4.	Fe_3_O_4_@ poly(lactic-co-glycolicacid) (PLGA)-PEG@ folic acid/curcumin	Targeted drug delivery for cancer treatment;	Encapsulation;	High drug loading and delivery; high in vitro targeting efficiency for cervical carcinoma; in vitro induction of apoptosis and reduction of tumor cell proliferation;	[244]
5.	Fe_3_O_4_@ C/carboxymethyl cellulose/chitosan/diclofenac sodium	Controlled drug delivery;	In situ conjugation and subsequent electrostatic conjugation;	High drug-loading efficiency; pH sensitive drug delivery;	[245]
6.	Fe_3_O_4_@ dextran	−	Covalent binding via electron pairing;	−	[246]
7.	Fe_3_O_4_@dextran	Near-infrared (NIR) photothermal ablation of tumor cells;	In situ encapsulation;	In vitro biocompatibility; in vitro and in vivo tumor growth inhibition after NIR activation;	[247]
8.	Fe_3_O_4_@ poly ε acrylic acid-gelatin/hydroxyapatite/polycaprolactone	Bone tissue engineering scaffolds for hyperthermia cancer treatment;	Electrostatic interactions between the acrylic acid and positively-charged Fe3O4;	Characterisation of the magnetic behaviour for hyperthermia applications;	[248]
9.	Fe_3_O_4_/poly-L-lactide (PLLA) nanofibers	Bone tissue engineering;	−	In vivo evaluation on tibia defect rabbit model; computer tomography and histological investigations revealed higher bone-healing potential than conventional PLLA	[249]
